# The Melbourne Diabetes Prevention Study (MDPS): study protocol for a randomized controlled trial

**DOI:** 10.1186/1745-6215-14-31

**Published:** 2013-01-31

**Authors:** Nathalie Davis-Lameloise, Andrea Hernan, Edward D Janus, Elizabeth Stewart, Rob Carter, Catherine M Bennett, Sharleen O’Reilly, Benjamin Philpot, Erkki Vartiainen, James A Dunbar

**Affiliations:** 1 Greater Green Triangle University Department of Rural Health, Flinders University and Deakin University, PO Box 423, 3280, Warrnambool, VIC, Australia; 2 Department of Medicine, North West Academic Centre, The University of Melbourne, Western Hospital, Melbourne, Corner Eleanor & Marion Sts, 3011, Footscray, VIC, Australia; 3 Deakin Health Economics, Deakin Strategic Research Centre - Population Health, Faculty of Health, Deakin University, 221 Burwood Highway, 3125, Burwood, VIC, Australia; 4 Faculty of Health, Deakin University, 221 Burwood Highway, 3125, Burwood, VIC, Australia; 5 National Institute for Health and Welfare, Mannerheimintie 166, 00300, Helsinki, Finland

**Keywords:** Protocol, Randomized controlled trial, Type 2 diabetes, Prevention, Lifestyle, Intervention

## Abstract

**Background:**

Worldwide, type 2 diabetes (T2DM) prevalence has more than doubled over two decades. In Australia, diabetes is the second highest contributor to the burden of disease. Lifestyle modification programs comprising diet changes, weight loss and moderate physical activity, have been proven to reduce the incidence of T2DM in high risk individuals.

As part of the Council of Australia Governments, the State of Victoria committed to develop and support the diabetes prevention program *‘Life! Taking action on diabetes’* (Life!) which has direct lineage from effective clinical and implementation trials from Finland and Australia. The Melbourne Diabetes Prevention Study (MDPS) has been set up to evaluate the effectiveness and cost-effectiveness of a specific version of the Life! program.

**Methods/design:**

We intend to recruit 796 participants for this open randomized clinical trial; 398 will be allocated to the intervention arm and 398 to the usual care arm. Several methods of recruitment will be used in order to maximize the number of participants. Individuals aged 50 to 75 years will be screened with a risk tool (AUSDRISK) to detect those at high risk of developing T2DM. Those with existing diabetes will be excluded. Intervention participants will undergo anthropometric and laboratory tests, and comprehensive surveys at baseline, following the fourth group session (approximately three months after the commencement of the intervention) and 12 months after commencement of the intervention, while control participants will undergo testing at baseline and 12 months only.

The intervention consists of an initial individual session followed by a series of five structured-group sessions. The first four group sessions will be carried out at two week intervals and the fifth session will occur eight months after the first group session. The intervention is based on the Health Action Process Approach (HAPA) model and sessions will empower and enable the participants to follow the five goals of the Life! program.

**Discussion:**

This study will determine whether the effect of this intervention is larger than the effect of usual care in reducing central obesity and cardiovascular risk factors and thus the risk of developing diabetes and cardiovascular disease. Also it will evaluate how these two options compare economically.

**Trial registration:**

Australian New Zealand Clinical Trials Registry ACTRN12609000507280

## Background

The dramatic increase in the prevalence of type 2 diabetes (T2DM) is posing a major international health problem [1] and there is an urgent need to implement a widespread and coordinated approach to its prevention. Worldwide, T2DM prevalence has more than doubled since 1981 and the total number of cases was almost 347 million adults in 2010 [2]. Currently in Australia, diabetes is the second highest contributor to the burden of disease, responsible for 5.2% of disability adjusted life-years (DALYs), and by 2023 it will be the leading cause, responsible for 8.6% of the overall disease burden [3]. Diabetes also poses an enormous economic burden, accounting for AU$1.4 billion in 2003 and projected to increase to almost AU$7 billion by 2033 [4].

Several recent clinical trials conducted on individuals have demonstrated that lifestyle modification with weight loss and moderate exercise can reduce the incidence of T2DM in high risk individuals by up to 58% [5-7]. Lifestyle modification has been shown to be even more effective than drug treatment using metformin or rosiglitazone [5,8] and seems to have an impact lasting at least several years following the active intervention [9-11]. The challenge is to implement the outcomes of these studies in the ‘real world’ of financially constrained health services.

In 2004 to 2006, the Australian Government funded a group-based lifestyle modification implementation trial - the Greater Green Triangle Diabetes Prevention Project (GGT DPP) - to determine its efficacy and feasibility in primary care [12]. From changes in waist circumference, it is imputed that this program reduced participants’ projected risk of diabetes by 40%, cardiovascular disease (CVD) by 16%. GGT DPP was derived from the Finnish Diabetes Prevention Study (DPS) clinical trial [13] and a sister project to the Good Ageing in Lahti Region (GOAL) Lifestyle Implementation Trial in Finland [14].

### Life! taking action on diabetes (Life!)

In Australia, the Federal and State Governments collaborate through the Council of Australian Governments (COAG). In health, diabetes prevention is considered one of the top priority areas. In 2007, as part of the COAG initiative for diabetes, the Victorian State Government committed AU$18.3 million of funding over four years for 25,000 high-risk individuals over 50 years old to participate in a diabetes prevention program called *‘Life! Taking action on diabetes’* (Life!) to be conducted by Diabetes Australia Victoria (DA Vic). Life! is a group based lifestyle change program and has direct lineage from the DPS, GOAL, and GGT DPP and its goals are described below.

The first Life! participants started the six session program in April 2008. At the start of June 2012, 13,946 people had commenced a Life! group course. In 2010, the Life! program underwent some changes to the entry requirements and intervention structure. The program will continue to evolve over time. In May 2011, the Victorian State Government announced continued funding for Life! for a further four years with an added emphasis on CVD prevention. The Life! program was originally accessible for individuals 50 years old and over with good English proficiency and at high risk of developing diabetes.

The goals of the Life! program were developed based on the experiences reported by two clinical trials of prevention of T2DM with life-style modification [5,6]. The intervention goals are: (1) no more than 30% energy from fat; (2) no more than 10% energy from saturated fat; (3) at least 15 g/1,000 kcal fiber intake; (4) at least 30 minutes/day moderate intensity physical activity; and (5) at least 5% reduction in body weight. Participants individually tailor and modify the goals over the course of the sessions.

### The Melbourne diabetes prevention study (MDPS)

The Melbourne Diabetes Prevention Study (MDPS) is a research project set up to study in detail a cohort of individuals undertaking the Life! program in order to evaluate the effectiveness of a large-scale prevention program and, importantly, the cost-effectiveness. The focus of the present study is a specific modification of the program developed in 2011. The program to be evaluated consists of an individual session and five group sessions (‘1+5 model’) as presented further below.

The working hypotheses of the MDPS are that (1) the MDPS program results in statistically and clinically significant changes (relative to usual care) in clinical, behavioral, and patient relevant outcomes, particularly changes in weight and waist circumference, that reduce the risk of progression to T2DM; and (2) the diabetes prevention program provided in this study is both ‘cost-effective’ (relative to usual care using a yardstick of $50,000 per quality adjusted life year (QALY)) and performs well in the second stage filter implementation analysis (the protocol for the economic appraisal will be presented separately).

## Methods/design

### Study design

The MDPS is a prospective, open, randomized controlled trial to assess the effectiveness and cost-effectiveness of a structured diabetes prevention program implemented in Victoria for people 50- to 75-years old who are at high risk of developing T2DM. This is a parallel group study, with the intervention group receiving a diabetes prevention program for 12 months and the control group receiving usual care from their General Practitioners (GPs) during the same time period. Intervention participants will undergo anthropometric and laboratory tests, and comprehensive surveys at baseline, following the fourth group session (approximately three months after the commencement of the intervention) and 12 months after commencement of the intervention, while control participants will undergo testing at baseline and 12 months only.

The study is coordinated from the Melbourne metropolitan campus of Deakin University (Victoria, Australia) and is supported by the National Health and Medical Research Council of Australia (NH&MRC). Ethical approval was obtained from the Deakin University Human Research Ethics Committee (EC66-2009). Written informed consent will be obtained from all participants.

MDPS uses the existing infrastructure and accredited Life! providers set in place by DA Vic. Deakin University is one of the accredited Life! providers. Life! facilitators will undergo additional training because the MDPS intervention is a ‘1+5 model’ version of the Life! program.

### Participants

The study population is comprised of individuals 50- to 75-years old, living in the Melbourne metropolitan area, who are at risk of developing diabetes, but currently do not have diabetes.

### Recruitment processes

Participants are recruited as described below from general practices, by accredited Life! facilitators, in pharmacies, and also at local community events within the study catchment. The project coordinator provides recruiters with specific training to ensure standardized recruitment procedures.

1) Recruitment through general practices

Patients 50- to 75-years old are invited to complete the Australian Diabetes Risk Assessment tool (AUSDRISK). Those who score 15 or more are referred to the study by their General Practitioner or Practice Nurse.

2) Recruitment through accredited Life! providers

A Life! provider is an organization accredited to deliver the Life! program. These organizations and DA Vic have the opportunity to refer individuals into MDPS.

3) Recruitment through pharmacies

MDPS project pharmacists and research assistants visit pharmacies and target screening to patients 50-years old and older who have easily-identifiable characteristics (such as high body mass index or blood pressure) that are suggestive of higher risk of T2DM. The questionnaire is completed in the pharmacy along with the individual’s details.

4) Recruitment through community organization or event

Recruitment also takes place in community settings such as health clubs/gymnasiums, local chapters of community service organizations (for example, Rotary, Lions Club and so on) and community fetes and expos.

### Eligibility

Individuals 50-to 75-years old are asked to complete an AUSDRISK test [15] that identifies individuals at high risk of developing T2DM within five years. Approximately one person in every seven with a score over 15 will develop diabetes within five years. The ten-item self-report AUSDRISK test is comparable with the Finnish Diabetes Risk Score (FINDRISC) tool [16], but includes ethnicity and country of birth appropriate for the multicultural Australian population.

**Figure 1:**
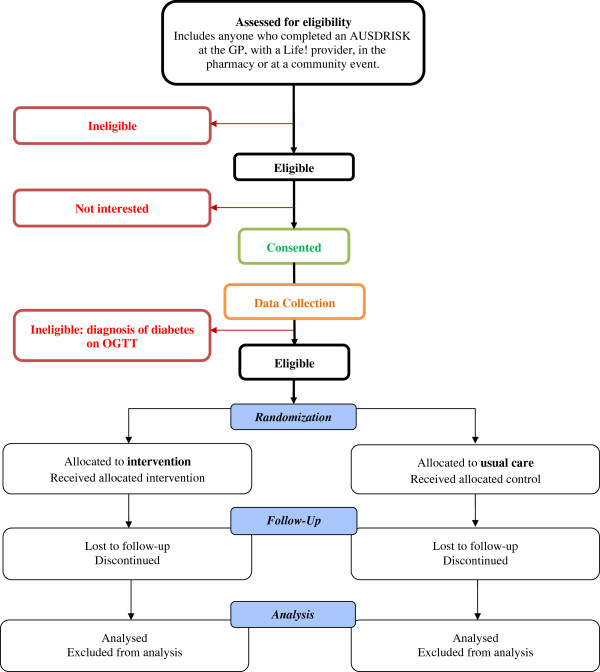
Flow chart of the MDPS.

The procedure for screening, eligibility and recruitment can be viewed in Figure 1. A score of 15 or more on the AUSDRISK test [17] is the primary inclusion criterion. If the score is 15 or above, the MDPS team member will assess the eligibility of the individual by checking the exclusion criteria, which for both males and females are (1) already on treatment for type 1 or type 2 diabetes; (2) laboratory evidence of existing T2DM defined as having a two-hour oral glucose tolerance test (OGTT) result of greater than or equal to 11.1 mmol/L and/or a fasting blood glucose greater than or equal to 7 mmol/L; (3) cancer (not in remission); (4) severe mental illness; (5) substance abuse; (6) myocardial infarction in the last three months; (7) pregnancy; (8) difficulty with English and (9) other household members involved in the study.

### Consent

The MDPS project coordinator contacts eligible participants to discuss the study, confirm eligibility criteria and arrange baseline testing. Eligible individuals recruited through the above strategies are provided with a plain language statement and consent form approved by the Ethics Committee at the time of recruitment.

### Intervention

The MDPS intervention consists of an initial individual session followed by a series of five structured group sessions. The initial individual session forms a specific design component of the intervention to maximize participant retention and increase their personal risk awareness. The delivery of an individual session runs in accordance with trials that have indicated that lifestyle interventions that incorporate individual feedback and goal setting foster a greater sense of participant engagement, motivation and ability to maintain lifestyle changes in the long term [11]. The individual session will be 30 to 45 minutes in duration, involving the participant and the facilitator only, and encompassing risk perception, goal setting, and motivation to change.

Four group sessions are provided at two week intervals. A fifth and final group session occurs eight months after the first group session. All sessions are led by a trained Life! facilitator, with generally 8 to 15 participants in each group. The duration of each group session is approximately 1.5 hours. The control group continues with usual care as provided by their GP. They may be offered a diabetes prevention program at the end of the study.

**Figure 2:**
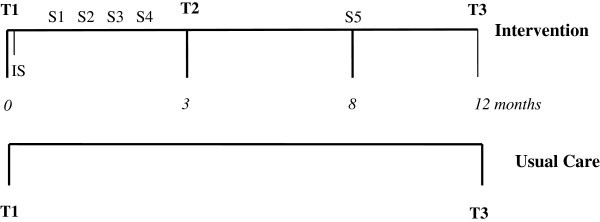
**Structure of the MDPS Group Program.** IS: Individual session.S1-S5: group sessions.T1, T2, T3: anthropometric and laboratory tests.

The intervention and data collection time points for both intervention and usual care groups are shown in Figure 2.

The theoretical framework of this intervention is based on the Health Action Process Approach (HAPA) model and self-regulation theory [18-20]. Several other theories, strategies and constructs are incorporated into the design of the intervention. These include the social learning theory, the trans-theoretical theory of stages of change [21,22], empowerment-oriented counseling [23-25], goal-setting approach [26,27], self-efficacy and self-evaluation [18,28].

The group sessions follow the structure of the HAPA model. Each session is structured with a major emphasis on creating an interactive and socially-supportive atmosphere. In these sessions the facilitator enables goal setting, moderates the discussion and feedback, and strengthens the role of the group as a source of social support. In addition, individual feedback is provided to participants from anthropometric and laboratory measurements at the second clinical testing session (which follows the fourth group session - approximately three months after the commencement of the intervention). This individual feedback is used to evaluate the impact of the intervention and to sustain participants’ motivation in their lifestyle changes.

### Outcome assessments

#### Primary outcomes

The primary outcomes under investigation are changes in diabetes and CVD risk as determined by changes in weight, waist circumference, fasting plasma and two-hour glucose, blood pressure and lipids. These are determined through anthropometric and laboratory measurements (performed as detailed below) at baseline, following the fourth group session (approximately three months after the commencement of the intervention) and 12 months after commencement of the intervention. The reduction in diabetes risk is calculated from the reduction in weight and waist circumference [12] and the reduction in cardiovascular risk from changes to the Framingham risk score, which incorporates the changes in the individual risk factors.

#### Secondary outcomes

Changes in psychosocial and quality of life measurements (performed as detailed below) are the secondary outcomes.

There is also an economic assessment of the program (separate protocol) by evaluating: whether it is ‘value for money’ through Cost Utility Analysis (CUA), evaluating technical efficiency issues through Cost Effectiveness Analysis (CEA) and by assessing a broader range of factors to supplement the technical analysis, based on second stage filter implementation analysis pioneered in Assessing Cost-Effectiveness (ACE) projects [29]. The final aim is to evaluate the ‘usual care’ of individuals at high risk of progression to T2DM.

### Anthropometric measurements and laboratory testing

Study nurses are specifically trained to undertake standardized anthropometric measurements including height, weight, waist and hip circumferences, and blood pressure. These measurements and the laboratory procedures follow the international recommendations: the World Health Organization’s Multinational Monitoring of Trends and Determinants in Cardiovascular Disease (MONICA) Protocol [30] and the latest recommendations of the European Health Risk Monitoring protocol [31]. All measurements are recorded on the Clinical Test Form and are performed at baseline, following the fourth group session (approximately three months after the commencement of the intervention) and 12 months after commencement of the intervention for the intervention group and only at baseline and 12 months for the control group.

### Anthropometric measurements

Height is measured at baseline and confirmed at subsequent testing using a portable height stadiometer (Charder). Participants are asked to remove their shoes and any hair ornaments. Height is recorded to the resolution of the height rule (1 mm).

Weight is measured with a weight scale (Charder MS3200) placed on a hard surface. The scale is tested in order to check that it gives a zero value. Participants are asked to remove their heavy outer garments and shoes, empty their pockets and remove heavy belts and items. The participant stands in the center of the platform, with weight evenly distributed between both feet. Weight is recorded to the resolution of the scale (0.1 kg).

The waist circumference is measured with a measuring tape (Seca203). The length of the tape is checked every month against the height rule. If the measuring tape is stretched it is replaced. Waist circumference is measured at a level midway between the lower rib margin and the iliac crest with a tape around the body in a horizontal position. Participants are asked to remove their clothes, except for light underwear. Measurements are recorded twice according to the resolution of the tape (1 mm) and the mean is used for data analysis.

Hip circumference is measured as the maximal circumference over the buttocks. The measurement procedure is the same as for the waist measurement with the exception of the tape position.

Blood pressure is measured with an electronic sphygmomanometer (Omron HEM-907). Blood pressure will be measured in the sitting position after at least five minutes rest. The measurement is taken from the bare right upper arm. The arm should be resting on the desk so that the antecubital fossa is level with the heart and the palm of the hand is facing up. The cuff should be placed on the right arm and its bottom edge should be 2 to 3 cm above the antecubital fossa. The top edge of the cuff should not be restricted by clothing. Two measurements are taken one minute apart and the mean is used for data analysis. If the second measurement differs by more than 10 mmHg systolic or 6 mmHg diastolic, a third measurement is taken one minute later. The mean of the closest two systolic and diastolic measurements is used for analysis.

### Laboratory measurements

Participants are asked to fast from 10 pm the night prior to their appointment and samples are drawn for fasting plasma glucose (FPG), HbA1c and lipid profile at baseline, following the fourth group session (approximately three months after the commencement of the intervention) and 12 months after commencement of the intervention and only at baseline and twelve months for the control group. Compliance with fasting is recorded before each clinical test by asking participants the duration of their fasting period. A two-hour OGTT follows in which the participant ingests a 75 g glucose solution. Two hours after this a further blood sample for glucose is taken. The time of the glucose ingestion and second blood withdrawal are recorded. The OGTT is performed at baseline and 12 months for both intervention and control arms.

The baseline results from the FPG and two-hour OGTT determine the final eligibility. If diabetes is diagnosed (FPG greater than or equal to 7 mmol/L and/or two-hour glucose greater than or equal to 11.1 mmol/L), the individual will be referred to their GP and will not be eligible to enter the study.

All patients who complete the clinical testing receive a letter documenting their results, with standardized feedback. A copy is also sent to their nominated GP. If diabetes is diagnosed, an individual covering letter is sent to the participant referring them back to their GP for suitable treatment.

Additional blood from consenting participants will be drawn and bio-banked at −70°C (ULT REVCO 1786) for further possible biochemical analysis.

### Psychosocial and quality of life measurements

Self-reported questionnaires are given to participants at baseline, following the fourth group session (approximately three months after the commencement of the intervention) and 12 months after commencement of the intervention. Health status questions include demographics, smoking status, history of diabetes, myocardial infarction, cancer and mental disorders. Self-regulation [32], self-efficacy specified for diet and physical activity [18,19,33,34], risk perception for diabetes, lifestyle planning, social support [35], and quality of life (AQoL8D) [36] are assessed. The Hospital Anxiety and Depression Scale (HADS) [37], the health related hardiness [38] and a physical activity assessment (Active Australia) [39] are also part of this questionnaire. Food Frequency Questionnaires (FFQ) [40] are completed at baseline and 12 months. Participants are also asked to complete a questionnaire related to the evaluation of the MDPS intervention at the 12 month clinical test.

If a participant scores 11 or more on the HADS tool for anxiety and/or depression at any of the testing sessions, an individual covering letter is sent to the participant referring them back to their GP for suitable treatment.

### Sample size and power calculation

For the estimation of sample size for two groups, with a two-sided 5% significance level and 80% power, the total number required will be 598 (299 in each arm). The sample size calculation was based on the observed mean change in diastolic blood pressure, a CVD risk factor, in the GGT-DPP and powered to detect an effect size of at least 0.23. To allow for an estimated attrition of up to 25% (estimate based on the GGT DPP), we require a total sample of 796 (398 in each arm). This sample size will provide sufficient power for other CVD risk factors, such as total and high-density lipoprotein (HDL)-cholesterol, as well as diabetes risk factors including fasting plasma glucose, weight, and waist circumference, as these are all expected to have larger effect sizes.

### Randomization

Randomization to either the intervention or usual care arm is made after confirmation of the eligibility of the patient, and after exclusion of T2DM, based on the baseline clinical test results (both FPG <7 mmol/L and OGTT two-hour glucose <11.1 mmol/L). Randomization is generated by a random number table and participant instructions are placed in individual sealed, opaque envelopes. The Project Coordinator selects the next sequential envelope to be attributed to a new participant. A covering letter is sent to participants indicating which group (Intervention or Control) they are allocated to, and what the subsequent processes are for participating in the study.

### Data management

Data are entered on two separate password protected databases: the ‘participant’ database and the ‘intervention’ database. The ‘participant’ database contains data on participants who have been registered in the trial along with their personal details, recruitment method, baseline testing date, allocations, and recall date for testing. This database is hosted on a secure, virtual server and is accessible only by the Project Coordinator, Study Nurse, Facilitator, administrative staff and Senior Program Manager. The second database, the ‘intervention’ database, contains de-identified data including blood test results, questionnaires, and anthropometrics. Data collected from participants at each testing session (baseline, following the fourth group session (approximately three months after the commencement of the intervention) and 12 months after commencement of the intervention) are checked for accuracy and completeness through routine data cleaning and management procedures.

The establishment of two databases allows management of the trial without violation of privacy or confidentiality of participant data and the integrity of allocation concealment. Only the Project Coordinator and specified members of the Study Team have access to identifiable data on a predetermined ‘need-to-know’ basis. The ‘participant’ database supports the regular generation of reports on those participants due for clinical testing, or completing an intervention.

### Data analysis

Analyses will be performed using Stata version 12 or later, and the ‘intention-to-treat’ principle will be adhered to. Baseline characteristics will be compared between intervention and control groups using chi-square tests, independent t-tests, and the Wilcoxon rank-sum test as appropriate. Primary and secondary outcomes (as described above) will be evaluated using mixed models, treating ‘group’ as a between-subject factor and ‘time’ as a within-subject factor. Two-sided tests will be used, with a level of *P* <0.05 determining statistical significance.

A separate and parallel economic evaluation protocol will be described elsewhere.

## Discussion

This study is necessary because it is important to know if this specific modification of the Life! diabetes prevention program leads to a reduction in diabetes risk, whether this reduction is larger than the effect of usual care and how these two options compare economically. Information about the characteristics of participants that predict completion of the program and improvement in clinical and behavioral measures will be useful for further development of diabetes prevention programs. Translating clinical trials into effective population programs is very challenging, but this translational process can be successful. We expect that MDPS will confirm the effectiveness of Life! and may allow us to refine it further.

The links to health policy are immediate: the COAG has on its agenda the prevention of progression to diabetes among those at high risk. The results of this study, including economic evaluation information, will inform policy and future guidelines and will complement the European recommendations for prevention of T2DM [41,42].

## Trial status

Recruitment of the participants started in September 2011. By 15 November 2012, 2,143 individuals had been assessed for eligibility and 266 had been randomized. Twenty-five individuals who completed baseline testing were found to have met T2D diagnostic criteria and were excluded from the study. It is anticipated that recruitment of participants will be end in February 2013.

## Abbreviations

AUSDRISK: Australian Type 2 Diabetes Risk Assessment; COAG: Council of Australian Governments; CVD: cardiovascular disease; DA Vic: Diabetes Australia Victoria; DPS: diabetes prevention study; FPG: fasting plasma glucose; GGT DPP: Greater Green Triangle Diabetes Prevention Project; GOAL: Good Ageing in Lahti Region; GP: general practitioner; HADS: Hospital Anxiety and Depression Scale; HAPA: Health Action Process Approach; MDPS: Melbourne Diabetes Prevention Study; OGTT: oral glucose tolerance test; T2DM: type 2 diabetes

## Competing interests

The authors declare that they have no competing interests.

## Authors’ contributions

EJ, RC, EV and JD were responsible for the research question, designed the study, and were responsible for obtaining funding for the study. NDL and AH wrote the first draft of this manuscript and with EJ were responsible for the revisions. CB, SO and ES contributed to specific sections of the manuscript. BP is the statistician and performed the power calculation, the sample size considerations, offered advice, and wrote the statistical analysis. JD is the general supervisor of the study and was involved in revising the article. All authors read and approved the final version of the manuscript.
